# Dynamic work trajectories and their interplay with family over the life course

**DOI:** 10.3389/fsoc.2023.1096109

**Published:** 2023-05-26

**Authors:** Xiaowen Han, Jeylan T. Mortimer

**Affiliations:** Department of Sociology, University of Minnesota, Minneapolis, MN, United States

**Keywords:** life course, intragenerational mobility, inequality, longitudinal analysis, work trajectories, work-family interplay, labor market

## Abstract

This review examines major bodies of literature, interrelated but usually considered separately, focused on work trajectories and their intersections with family dynamics through the life course. It begins with a consideration of the life course paradigm, which draws attention to the temporal dimensions of human lives, and recently developed analytic techniques that are well-suited to empirical investigation of life course transitions and trajectories over time. The review proceeds to examine empirical research on work career mobility (including both inter- and intra-generational mobility) measured as either trajectories of continuous outcomes or sequences of categorical outcomes, and their long-term consequences for socioeconomic attainment. Work-family trajectories are then addressed, focusing on the impacts of family on work, notably expressed in the motherhood wage penalty, and how family structure and processes affect long-term labor market outcomes. Research documents considerable heterogeneity in work-family dynamics over the life course across social groups with unequal resources. The review concludes with an assessment of the interplay of work and family trajectories studied longitudinally and makes recommendations for future research. It is argued that while extant studies of the work-family interface are compatible with, and sometimes deliberately reflect, a life course perspective, these bodies of research would benefit from more fully incorporating the life course principles of “agency” and “time and place”.

## 1. Introduction

During the past several decades, profound changes have engulfed labor market systems, reshaping individual work lives in many societies, particularly in the global North. The hallmark of these changes is uncertainty or instability. In the realm of employment across the US, breakup of Fordist production and labor processes since the early 1970s has inaugurated a new regime of “flexible” capitalist accumulation and a correspondingly “flexible” mode of labor regulation (Harvey, 1989). Stable, secure employment with benefits, once promised in the Fordist employment regime, has been replaced by the proliferation of precarious work that is “uncertain, unstable, and insecure” (Kalleberg and Vallas, 2018). Structural shifts have fostered employment instability and precarity, including de-unionization (Western and Rosenfeld, 2011), financialization led by the growing power of institutional investors and Wall Street (Ho, 2009), geographic mobility of capital in the era of globalization, and the digital revolution fueling the growth of the “gig” platform economy (Srnicek, 2017). Macro-level crises, such as the Great Recession and the ongoing global COVID pandemic, have exacerbated the precarity of paid work. In the European context, while precarious work has also become an important feature of socioeconomic insecurity, macro-level factors shape great variation in the distribution of precarious work across countries (Mai, 2017). Many of the changes toward instability in the labor market have been paralleled by similar trends toward flexibility and instability in the domain of family, the private spheres of social lives, as seen by the increasing rates of union dissolution and declining marriage and fertility levels (Cherlin, 2004; Lesthaeghe, 2010). Many have attributed such demographic trends, at least partially, to women's participation in paid employment and their economic independence (Becker, 1991; Oppenheimer, 1994; Goldscheider et al., 2015).

While macro-level descriptions based on cross-sectional statistics have alluded to individual biographical variation across life stages, such snapshot reports fail to capture potentially long-term dynamic changes across individual work trajectories and family lives. As C. Wright Mills remarked in his classic book *The Sociological Imagination*, “no study that does not come back to the problems of biography, of history, and of their intersection within a society has completed its intellectual journey” (Mills, 1959). In response to Mills, life course research aims to better understand interlocking biographical trajectories across multiple domains within and across specific macro-structural contexts. With the increasing availability of longitudinal data, either locally or nationally representative, and sometimes internationally comparable, life course social scientists have made substantial progress in developing innovative methods to capture work trajectories and the interplay between work and family as interlocked multidimensional processes, with special interest in how their patterns and consequences are shaped by inequality. In this review, we summarize recent empirical findings on the work trajectories and their interplay with family lives. The scope of this review is for the most part limited to North American and European contexts due to data availability and space constraints. We argue that further understanding of social disparities in work trajectories and their intersections with particular family dynamics would result from further incorporation of the life course perspective.

The review proceeds as follows. Section 2 presents a brief theoretical overview of life course sociology. We introduce basic life course concepts and methods that elucidate biographical trajectories. Section 3 provides an empirical review of research on work trajectories, distinguishing studies that model the work life course as a trajectory of continuous outcomes (e.g., income) from those modeling sequences of categorical work outcomes (e.g., employment status). While empirical studies of work careers sometimes overlook the interdependence of work and family domains and the joint consequences of trajectories in both spheres, attention to these linkages has been growing, as documented in Section 4. There we consider research on intertwined work and family lives, extending from studies that only address contemporaneous or short-term impacts of one domain on the other to recent studies with a life course emphasis that examine the interplay of work and family trajectories across the life span. In the last section, we draw attention to research gaps in extant literature and the yet unrealized potential of the life course perspective to promote further understanding of work trajectories and the work-family interface.

## 2. Life course paradigm

### 2.1. The emergence of the life course paradigm and its principles

The life course paradigm refers to a cohesive set of principles, concepts, and methods that guide research on human lives over time and across changing contexts (Elder, 1998; Shanahan and Macmillan, 2008). Instead of examining static outcomes, the life course paradigm is “dynamic” in nature, depicting individual biography in changing and processual terms. The publication of *Children of The Great Depression* (1974), by Glen H. Elder Jr., marked a watershed in life course sociology, tracing the consequences of family economic crisis during childhood and adolescence for adult education, work, and family outcomes among two adjacent cohorts of youth. In the following decades, numerous empirical studies in sociology have followed Elder's groundbreaking approach to investigate the effects of the “past” on the “future” life outcomes of individuals and their variations across social groups and contexts (Elder, 1974). Building upon these early empirical endeavors, Elder et al. (2003) identified five principles of the life course paradigm. **First**, according to the principle of life-span development, human development does not end at a certain stage in life but involves fundamental biological, psychological and social changes from birth to death. Importantly, earlier events and developmental states influence subsequent progressions. **Second**, the principle of time and place recognizes that the individual life course is “embedded and shaped by the historical times and places they experience over their life” (Elder et al., 2003). **Third**, the principle of timing, or the “life-stage principle”, states that the meaning and consequences of events depend on when they occur in a person's life. **Fourth**, the principle of linked lives recognizes that human lives are interdependent; the effects of social change on people's lives depend on their network of interpersonal relationships. **Fifth**, the principle of agency highlights the active roles individuals play in constructing their own life courses through their actions, reactions, and choices. Furthermore, the multidimensionality of lives, including parallel and interdependent trajectories in work, family, and health, is occasionally highlighted as an additional sixth principle (Fasang and Mayer, 2020).

While these general “paradigmatic principles” from Elder and colleagues have been indispensable in establishing life course sociology, Bernardi et al. (2019) have built on this early theoretical foundation to propose a “life course cube” that offers a more integrated view of life courses processes. As a synthetic representation of the life course, the cube has three axes referencing “three dimensions of time, life domains, and levels at which developmental, behavioral and social process occur” (Bernardi et al., 2019, p. 2). The time axis features the interdependence of historical, present, and future life course processes. The life domains axis recognizes that individual goals, resources, and behaviors are interrelated across domains, including elements of the work-family interplay at the core of the current review. The multilevel interdependence of the life course connects individual action and behavior with the life courses of other people (“individual action level”), the external social structure (“supra-individual level”), and internal dispositions and psycho-physiological functioning (“inner-individual level”). The crossing of the cube's axes pinpoints crucial nodes of interaction within an overall structure of life course interdependence.

### 2.2. Life course concepts and models

Empirically, the life course can be conceptualized as the “age-graded sequences of roles, opportunities, constraints, and events that shape the biography from birth to death” (Shanahan and Macmillan, 2008). The time axis, whether defined by historical context, birth cohort, or individual age, is central to modeling the age-graded life course. Social pathways, the “trajectories of education and work, family and residences that are followed by individuals and groups through society” (Elder et al., 2003), define how individual lives are socially organized and structured by historically situated forces and social institutions. For example, paid work and career pathways, institutionalized in the middle of the 20th century for middle-class white-collar and unionized blue-collar men in the U.S., provided a template for age-graded labor market entry and exit and advancement through internal career ladders; these are no longer available to most contemporary workers (Moen, 2016a). Trajectories, or sequences of roles and experiences, are themselves made up of transitions or changes in state or role. Role entry and exit mark each transition, which is embedded in a trajectory that gives it specific form and meaning. The trajectory can be charted temporally by linking institutionally defined role transitions with a defined sequence, duration, and order. The meaning of a transition depends on its place in a trajectory, consistent with the life-stage principle of timing. Some transitions, called “turning points”, have the potential to produce dramatic change in both the internal and external aspects of the life course (Shanahan and Macmillan, 2008). The degree to which trajectories and transitions “correspond or deviate from social expectations” may be “consequential for life chances” (Macmillan and Eliason, 2003). A large body of research investigates the timing and order of transitions, or the sequencing of social roles, in work and family. Questions often address subgroup variations in the determinants and consequences of these transitions or trajectories within or across life domains, as these contribute to the understanding of stratification and inequality (Shanahan, 2000; George, 2003).

Along with the emergence of high-quality longitudinal panel data that trace respondents over time, statistical models have been developed to capture the variation and complexity of the individual life course. These methods can be generally categorized as event-based or holistic (Billari, 2005). Event-based approaches focus on the timing and/or occurrence of particular events or transitions (for example, transition from school to work or transition to parenthood) and their causes or correlates. Event history analysis examines how covariates of interest influence individuals' probability (“risk” or “hazard”) of experiencing certain events or transitions (Wu, 2003; Allison, 2014; Mikolai and Amos, 2020), such as marriage, parenthood, or divorce in the family domain, and obtaining a first job, job displacement or unemployment, and retirement in the domain of paid work.

A more holistic approach develops algorithms to describe the totality of trajectories, especially in the form of sequences of transitions in either single or across multiple domains (Pollock, 2007; Mayer, 2009; Gauthier et al., 2010). Statistical models developed in recent decades adopt this holistic approach in modeling age-sequenced trajectories of individual attributes, statuses, or roles (usually measured as categorical outcomes). These include optimal matching analysis (or sequence analysis, Abbott, 1995; Ritschard, 2021; for a recent review see Liao et al., 2022), and finite-mixture models such as latent class analysis (LCA) and group-based trajectory models (GBTMs) (George, 2009; Nagin, 2014). The main goals of these trajectory-based models are first, to identify a finite number of discrete trajectories in a population to arrive at typologies of the life course; second, to assign individuals to a specific trajectory group; and third, to relate the trajectory group assignment to individual characteristics or outcomes. A recent methodological advance proposes the combination of sequence analysis and event history analysis to better understand the effects of time-varying covariates on the transition from a state to ideal-typical trajectories (Studer et al., 2018). As for continuous outcomes like wage or occupational prestige, growth curve models appear to be useful tools for modeling change and volatility (George, 2009; Mayer, 2009).

The choice of analytic models depends on the measurement of the variables of interest in different life domains. Work life variables can be metric (e.g., occupational prestige or earnings) or categorical (e.g., employment, unemployment, or out of the labor force). Family trajectories are usually represented as sequences of categorical states (e.g., single, married, divorced, remarried). While much research relies on quantitative measures, life course researchers have demonstrated the value of qualitative methods, or mixed approaches including both qualitative and quantitative data, for in-depth understanding of life course trajectories (Damaske, 2013; Le Roux et al., 2023).

## 3. Work trajectories over the life course

### 3.1. Shifts in mobility research: from a static to a dynamic approach

Sociologists' interest in the sources and extent of inequality motivates their attempts to describe and explain how people are allocated to unequal positions in the stratification system, mainly through the paid labor market. The social mobility of individuals is traced both across and within generations (Kalleberg and Mouw, 2018). *Inter*generational mobility, measured as the resemblance of “origin” status (parents) and “destination” status (adult children), attracts more attention from mobility scholars, due to its centrality to the understanding of (in)equality of opportunity or openness of the class structure. By contrast, *intra*generational mobility refers to “changes in individuals' economic positions or occupational standing over their working lives” (Kalleberg and Mouw, 2018). Though it receives less scholarly attention than intergenerational mobility, intragenerational mobility is of considerable importance in understanding inequality processes across the life course (Cheng, 2014; Jarvis and Song, 2017).

Intragenerational career mobility refers to transitions among jobs and occupations, or changes in earnings and other quantifiable job rewards through the individual career (Kalleberg and Mouw, 2018). Variation in intragenerational mobility is due to individual attributes (both ascribed and achieved characteristics) and structural factors at both meso-institutional (i.e., organizations, firms, industries) and macro-societal levels (i.e., state interventions). As for individual attributes, economic theories are often cited to explain divergent patterns of intragenerational mobility, such as the human capital model (Mincer, 1957; Becker, 1964) and theories of discrimination (Becker, 1957; Cain, 1986). Focusing on the supply-side of the labor market, human capital theory argues that individuals invest in themselves through education, experience, or various forms of training to increase their productive capacities, fostering occupational and wage attainments in the long run (Goldthorpe, 2014). To a certain extent, the economists' assumption of individuals as rational actors who make informed choices about their human capital investment resonates with life course scholars' emphasis on agentic power. However, external factors like discrimination against racial minorities and women in education and the labor market prevent the full exercise of human agency, diminishing the return to human capital. Arguments stressing the demand-side of the labor market emphasize the role of structural forces in career mobility, stemming from firms/organizations, occupations, industries, and macro-societal economic contexts. The internal labor market within firms is key to the structural side of the story (Rosenfeld, 1992).

### 3.2. Life course trajectories of intragenerational career mobility

With the increasing availability of panel data collected over long periods of time, researchers have become increasingly innovative in constructing measures of key dimensions of intragenerational mobility, including transitions, sequences, durations, and trajectories. Some model mobility trajectories of continuous job rewards like wage or income (Cheng, 2014, 2021) or occupational prestige (Lersch et al., 2020). Others emphasize trajectories of career mobility by depicting sequences of categorical outcomes of work like employment status (Muñoz-Bullón and Malo, 2003; Manzoni et al., 2014; Vuolo et al., 2014; Weisshaar and Cabello-Hutt, 2020; Van Winkle and Fasang, 2021). Transitions in the career are depicted by event history analysis, such as entry into the labor market from school (Kerckhoff, 2000; Becker and Blossfeld, 2017), or transitions into and out of employment (DiPrete, 1981; Mont'Alvao and Ribeiro, 2020) and low-wage precarious jobs (Schultz, 2019).

#### 3.2.1. Trajectories of continuous measures of work

Longitudinal surveys that collect long-term individual work history data allow construction of individual trajectories along the continuous measures of work indicators. Some well-examined continuous measures of work include wage or earnings from paid employment and occupational prestige scores. Empirical studies have documented divergent earnings trajectories across gender, race, and education, suggesting that less advantaged social groups tend to have lower starting levels as well as slower growth rates of earnings. Moreover, the intersectionality of social inequality exacerbates the life course inequality of earnings trajectories for social groups with multiple dimensions of disadvantage.

Extending cross-sectional findings of wage inequality across social groups, most prominently gender and race gaps, studies have found persistent and even magnifying subgroup inequality over the individual life course (Rosenfeld, 1980), the latter supporting the cumulative inequality hypothesis (Dannefer, 2003). As noted earlier, a useful analytic technique to assess vertical mobility is the growth curve model, permitting analysis of long-term careers. Cheng (2014) applies the growth curve model framework to decompose intracohort life course inequality of hourly wage into three parts: (1) random variability (within-person random fluctuations), (2) trajectory heterogeneity (variations in baseline wage and wage growth rate), and (3) cumulative advantage between and within social groups (the covariance between baseline and growth rate). Using longitudinal data from the National Longitudinal Survey of Youth−1979 Cohort (NLSY79) to empirically examine these three properties, Cheng finds that social groups defined by gender, race, and educational attainment significantly differ from each other in both baseline wage and growth rate. Compared to men, whites, and more highly educated individuals, women, blacks, and less-educated individuals have both lower baseline wages (intercept) and a slower growth rate (slope). The positive correlation between the two trajectory heterogeneity indicators indicates a pattern of cumulative advantage; those with an initial advantage not only maintain their advantage but also accrue more benefits as they grow older (Dannefer, 2003; DiPrete and Eirich, 2006).

Tomaskovic-Devey and Skaggs (2002) argue that the gender wage gap can emerge and intensify over people's careers due to a social closure process that excludes female workers from on-the-job training and productivity-enhancing workplace networks. Besides the disparities arising from male advantages in the workplace (Tharenou, 2013), crucial family transitions like marriage and parenthood produce disparate returns in the labor market for men and women. Tomaskovic-Devey et al. (2005) find that blacks and Hispanics have flatter wage trajectories than whites. They argue that race-based cumulative advantage is due to discrimination, social closure in the workplace, and depreciation of the human capital of racial minorities over their careers.

Finally, since people with higher educational attainment are usually thought to have a greater accumulation of human capital (Goldthorpe, 2014), they receive higher wages at entry to the labor market. They also experience faster wage growth due to positive feedback loops among motivation, resources, and promotion (DiPrete and Eirich, 2006). Applying the same analytic framework to data from the Panel Study of Income Dynamics (PSID), including respondents from different cohorts, Cheng (2021) finds that the cumulative wage advantage associated with higher educational attainment has increased across cohorts of white male workers born from the 1940s to the 1970s.

While group disparities are often examined as additive covariates in analytic models, recent studies have recognized the intersectionality of multiple dimensions of status that expose individuals to distinct combinations of (dis)advantage in processes of career mobility. Empirical evidence for a widening gender wage gap over time is found predominantly among college and advanced degree holders, where glass ceilings are most likely present, indicating that educational advantage intersects with male advantage to produce divergent earnings trajectories (Bertrand et al., 2010; Goldin, 2014). Dividing women into two educational groups, those with college education and those without, Alon and Haberfeld (2007) find disparate racial wage gap trajectories by age. They document constant racial wage gaps among women with higher education but a widening race gap among those without higher education. The authors argue that the deficit in labor market experience among black and Hispanic individuals plays a crucial role in creating divergent wage trajectories across racial groups for women without college education.

A different pattern is revealed for men. Tomaskovic-Devey et al. (2005) find that long-term racial earnings disadvantages of blacks and Hispanics are more prominent among the college educated than less educated men. Emphasizing the three-way interaction among gender, race, and education, Doren and Lin (2019) fit growth curve models of annual earnings separately for blacks and whites using data from NLSY79. Their findings provide evidence that holding multiple forms of gendered, racial, and/or educational advantage have interactive effects that accumulate across life. Particularly, the gender gap expands most with age for whites and the college-educated, where the male premium is compounded by racial and educational advantages.

Mixed effects and fixed effects models for analyzing longitudinal wage and earnings gap trajectories are flexible enough to incorporate job-related features, like the accumulation of work experience (Tomaskovic-Devey et al., 2005), and significant life events in the domain of family (DiPrete and McManus, 2000). For example, Tomaskovic-Devey et al. (2005) include job tenure as a proxy for human capital in their fixed effects analysis. They find that racial gaps in wage trajectories were partially mediated by racial disparity in human capital accumulation in the labor market.

Besides wages and earnings, other continuous measures of career mobility include occupation-level characteristics like average occupational education and income, and occupational prestige. Becker and Blossfeld (2017) incorporate both individual characteristics and structural changes to predict upward, downward, and lateral mobility along the occupational prestige scale among West German male workers. Consistent with human capital theory, they confirm that education promotes upward mobility and protects the worker from downward mobility; increasing labor market experience reduces the likelihood of downward prestige mobility. With respect to the broader economic context, the authors argue that modernization promotes career mobility in all directions. Applying the growth curve framework to decompose intracohort life course variance in occupational prestige in West Germany, Lersch et al. (2020) find that the cohorts that entered the labor market in the late 1950s and 1960s experienced less variability in both baseline wage and in wage growth (less trajectory heterogeneity) compared to earlier and later cohorts. They argue that internal labor markets and closed employment relations in the postwar era could partially explain the cohort difference in volatility of occupational standing. With respect to subgroup variation, Lersch et al. (2020) find that women in West Germany have higher volatility in life course occupational prestige than men, which could be attributed to life events interruptions as shown with their simulated data.

Manzoni et al. (2014) also use multilevel growth curve analysis to model change in occupational prestige among cohorts of West German respondents. They find persistent influence of class background on long-term occupational attainment, mediated by educational attainment for women but not for men. The authors find limited evidence for differential change across cohorts, in contrast to Lersch et al. (2020)'s findings of increasing volatility in prestige since the late 1950s.

Miech et al. (2003) use average occupational earnings and educational levels matched to NLSY79 respondents' occupational codes to model changing economic returns to occupation over time. Their findings reveal that the gender disparity in average occupational standing stays constant over the life course, as men score consistently higher than women in terms of occupational income but consistently lower in occupational education. But the occupational standings trajectories differ significantly across racial groups, as the occupational earnings gap between blacks and whites grow larger over the life course.

#### 3.2.2. Trajectories of categorical measures of work

Researchers have also studied movement across categorical work-related statuses that are not indicative of change in continuous inequality outcomes, though some studies examine the implications of such movement for indicators of upward or downward mobility like income or wealth. For example, transitions from employment to unemployment, and trajectories that feature long-term unemployment or underemployment, have negative consequences for earnings (DiPrete, 2002). Empirical studies have generally confirmed that trajectories featuring steady employment, which are mainly accessible to more advantaged social groups, have greater economic returns. Studies focusing on cross-national comparisons have constructed measures of trajectory complexity to examine the changing patterns of work across contexts (Van Winkle and Fasang, 2021). Methods for identifying patterns of such age-graded trajectories, such as sequence analysis or latent class analysis, simplify the study of individual career histories by reducing them to identified clusters.

Weisshaar and Cabello-Hutt (2020), using the percentage of employed weeks per year as a measure of attachment to the labor market, fit group-based trajectory models to identify six common employment trajectories between ages 22 and 50 for NLSY79 respondents. They find that women across racial groups and black men are more likely than white and Hispanic men to have non-steady employment trajectories and lower levels of employment throughout their lives. Individuals who have experienced poverty also have heightened risks of intermittent employment. The authors then associate the employment trajectories with wages later in the career, at ages 45–50, and confirm the hypothesis that the “steady higher employment trajectory” leads to the highest wages. Family and childcare responsibilities might explain why women are more likely to incur employment lapses than men, but the trajectories also differ across racial groups.

Killewald and Zhuo (2019) identify five employment patterns following the birth of a first child among women respondents in the NLSY79 over the first 18 years after maternity. Besides the effects of human capital, gender attitudes and family structure in channeling mothers into different employment patterns, the authors find that net of other covariates, black mothers have higher odds of membership in the full-time employment group and lower odds of being in the part-time group than white mothers (see also Lu et al., 2017). Findings in Italy yield similar gendered employment trajectories, as Struffolino (2019) demonstrates that Italian women, especially those with lower levels of education, are more likely than men to experience pathways characterized by instability, weak (or absent) employment, and less social security protection that ultimately lead to more precarious early careers. In the context of China, Lin (2013) identifies four clusters of 25-year career trajectories across broad categories of occupations. Men with higher education and political resources were more likely to be sorted into upward and stable trajectories.

From a comparative lens, Van Winkle and Fasang (2017) use sequence analysis to analyze both cross-cohort and cross-country variation in the complexity of employment trajectories in 14 European countries. Their composite measure of the number of transitions between different jobs and non-employment states over the career between 1933 and 2008 addresses the complexity of trajectories. While complexity increased across birth cohorts, differences in complexity were greater across countries than across cohorts, perhaps attributable to national institutional variation, such as the strength of school-to-work links and employment protection legislation. The authors then replicated and updated the analyses in 2021, including additional countries in Europe and more recent cohorts. The updated findings support a moderate increase in employment complexity that pale in comparison to large and stable cross-national differences for work lives experienced from 1934 to 2016 across 30 European countries. Other studies using clustering techniques focus on individuals employed in particular industries or occupations to trace career development trajectories, such as Joseph et al.'s (2012) description of heterogeneity in career paths among IT workers.

#### 3.2.3. Labor market transitions and their longitudinal implications

While many studies predict transitions over short time intervals, more relevant from a life course perspective is a line of research that examines the long-term influence of transitions on later wage or earnings trajectories. Hollister (2012) demonstrates that while some transitions, such as employer-only changes, may bring increased opportunities for wage increases, changes in career bring risks of large wage losses. Fuller (2008) examines the life course association between job mobility and wage trajectories in greater detail. Although workers who frequently switch employers generally end up earning less than their more stable counterparts, the type, timing, and level of changes strongly affect the ultimate wage differential. For example, involuntary job losses are detrimental but quitting a job voluntarily may boost wages; and early quits benefit wages more than quits that take place later in the career (Fuller, 2008). Moreover, differences in men's and women's labor-force attachment and family circumstances are also influential, as those who are less attached to the labor market benefit less economically from changing jobs (employers), and women who are married or have children also experience less favorable mobility and wage outcomes.

Frederiksen et al. (2016) draw on panel data from Denmark to examine the presumably positive effects of within- and cross-firm mobility on earnings growth. They find that cross-firm moves at the non-executive level provide sizable, short-run earnings growth. However, these gains appear modest compared to the persistent impact of promotions at the executive level both within or across firms on earnings growth and subsequent mobility. Another study in a comparative context confirms that job-to-job mobility rewards movers with positive outcomes in eight European countries, but the impacts of job mobility via unemployment are negative (Schmelzer and Ramos, 2016).

Using data from the PSID, Schultz (2019) examines the wage mobility of workers starting employment spells at low wages and finds that about half of low-wage workers move to better wages within 4 years. But the mobility rates are stratified by age, gender, education, occupation, and job characteristics. DiPrete and McManus (2000) term labor market and family transitions as events with the potential to “trigger” a change in a household's future income trajectory. They find that institutional contexts moderate the negative consequences of unemployment, as German social welfare policies shelter individuals from unemployment better than in the US (see also DiPrete, 2002).

## 4. Intertwined work and family trajectories over the life course

The complexity of the individual life course is revealed by interdependent trajectories in multiple domains of social life, such as education, work, and family (Bolano and Berchtold, 2021). Accumulation of resources in one domain may facilitate or hinder adaptation in another domain (Moen et al., 2013). The next section considers the intertwined relationships between work and family trajectories. While studies have generally found negative impacts of major family life events, particularly transition to parenthood, on labor market inequalities among women, the magnitude of the impacts varies across life course timing and duration, as well as across social groups with unequal socioeconomic resources. Further research on the diverse trajectories of intertwined family and work lives reveals persistent gender inequalities across societies with different levels of welfare policy support.

### 4.1. Family to work: the impacts of family life events on work lives

As shown in many studies documenting divergent career mobility trajectories for men and women, women face cumulative disadvantages. At entry to the labor market and in their growth trajectories of labor market outcomes over time, they have persistently lower wages, earnings, and occupational prestige, and more unstable employment. Important family life events, including union formation, dissolution, and parenthood interrupt women's work and lead to gender divergence in career progression.

The moderation of work careers via gender (dis)advantage could be illustrated in several ways, for example, by considering the age of marriage or the effects of divorce and repartnering on career advancement. Due to space limitations, this review focuses on the “motherhood penalty”, which has attracted considerable scholarly interest in recent decades. Based on the increasing availability of longitudinal survey data and the development of fixed-effects regression models, investigators have reached consensus that on average, mothers earn lower wages than childless women; by contrast, fathers obtain a premium in wages compared to childless men. While the precise magnitude and the mechanisms driving the penalty or premium are not fully agreed upon, several theories have been tested in the empirical literature; these highlight human capital, work effort, job characteristics, employer discrimination, and selection (Gough and Noonan, 2013). Recent studies have focused on heterogeneity in the penalty by age, race, marital status, parity, and socioeconomic status indicated by education and income (Budig and Hodges, 2010; England et al., 2016).

The above literature has well-documented the heterogeneity of the wage penalty associated with motherhood and has investigated the mechanisms underlying this effect. However, most empirical studies follow a rather “static” approach. That is, they typically construct a single measure of parenthood penalty based on a comparison between labor market outcomes like wages earned by mothers and non-mothers at a particular point in time, ignoring the possibility of temporal variation in the effect of parenthood across the life course. Relatively little research has examined the long-term effects of family transitions like entering marriage or parenthood on labor market trajectories over longer periods of time. From a life course perspective, such family life transitions are not one-time events, but major turning points that mark the beginning of a long-term life experience. As a result, the motherhood penalty may unfold gradually over the life course, subsequent to first or later births.

Kahn et al. (2014) interact age decade intervals with the number of children in fixed effects models to predict long-term trajectories of women's labor market participation, occupational status, and hourly wage since childbirth. The authors find that motherhood is “costly” to women's careers, but the effects on all three labor market outcomes attenuate with age. Children reduce women's labor force participation, but this effect is strongest when women are younger, and it is eliminated by the time they reach their 40s and 50s. Mothers also seem to regain ground in terms of occupational status over time. The wage penalty persists across the life course only for women who have three or more children. Doren (2019) demonstrates that the motherhood penalty for highly educated women varies across life stages, as those who postpone their first birth to the late 30s even receive a significant premium, presumably due to distinct human capital accumulation patterns across career stages.

Van Winkle and Fasang (2020) build on the analytic framework developed by Kahn et al. (2014) to assess variation in the motherhood penalty across the life course; they also extend the analysis to fathers, generating race and gender specific estimates. Fitting separate models to six intersectional gender-by-race groups (white, black, and Hispanic), the authors examine the varying effects of having different numbers of children over the life course. They find that only white women with three or more children suffer large and persistent motherhood penalties up to age 40, but black and Hispanic women, regardless of the number of children, experience penalties concentrated in a brief age range of 5–10 years around age 30. Fatherhood premiums, confined to brief periods in early adulthood, are only found among white men. Besides the family impacts on changing point-in-time earnings across the life course, studies have also looked into the divergent accumulation of earnings (or lifetime earnings) following different family life courses. Using register data from Finland, Jalovaara and Fasang (2020) highlight the accumulated earnings premium for Finnish men and women who follow the most normative family lives of stable marriage and parenthood, while mid-life earnings (accumulated from ages 18–39) are lowest for unpartnered mothers and never-partnered childless men.

Looking into couple dynamics, Musick et al. (2020) trace mothers' share of couple earnings in the 10-year window around the first birth using panel data from three industrial societies: the US, Germany, and the UK. The authors find steep declines in the mother's share of couple earnings following the first birth across the three societies. Such decline would likely maintain or even exacerbate motherhood wage disadvantage as couples with children would have less motivation to prioritize mothers' career prospects when making relevant decisions (e.g., decreasing women's hours of work, continuing education or vocational training, geographic mobility in favor of husbands' jobs, etc.). Decreasing women's share of couple earnings persists over several years of follow-up, though declines are smaller in the US due to higher female employment rates and longer work hours.

As it is an important transition, the timing of employment re-entry after childbirth has been another life course research focus in studying the labor market consequences of family life. Aisenbrey et al. (2009) examine the differences in the timing and consequences of employment re-entry after transition to motherhood among women in Germany, Sweden, and the United States. They find that US women have the shortest time-out periods after childbirth, as 75% of them are back at work only 6 months after the birth of a first child. However, consistently across the three countries, time-out periods following childbirth tend to destabilize women's careers and increase their risks of downward occupational mobility even when those periods out of the labor force are short.

Another line of research, also adopting a dynamic life course approach, aims to depict career trajectories following the motherhood transition over a longer time span using clustering models like sequence analysis. Hynes and Clarkberg (2005) apply a group-based trajectory method to examine women's employment trajectories across the period of early parenthood (12 months before and 24 months after childbirth) using data from the NLSY79. The authors find that throughout early parenthood, women exhibit significant movement into and out of the labor force. Factors that predict varying levels of motherhood wage penalty also predict different employment trajectories, with the more advantaged women channeled into the “continuously employed” trajectory. Moreover, the trajectories also differ by parity; overall employment rates are lower around the second birth than the first birth, with more mothers at second birth channeled into trajectories that feature lower attachment to the labor market.

Lu et al. (2017) adopt sequence analysis to chart the employment trajectories of women in the first year following childbirth using data from the SIPP. The authors find that most women who were employed before childbirth show a high degree of labor market continuity, but a notable share of them (24%) took less stable paths featuring frequent transition into and out of the labor force. They further demonstrate that the trajectories vary across racial groups and by nativity; non-white women exhibited greater labor market continuation than white women, and immigrant women with shorter length of U.S. residence were more likely to reduce employment than native born women. Killewald and Zhuo (2019) extend the observation window to cover the first 18 years of maternity and identify five common employment patterns of American mothers: three patterns revolve around a single employment status of either full-time (36%), part-time (13%), or non-employment (21%), while the other two patterns are characterized by 6 (15%) or 11 (14%) years of non-employment, followed by a period of transition back to employment.

### 4.2. The interplay of work and family trajectories

While the above studies focus on the unidirectional impact of family events on employment outcomes, and studies by family demographers routinely analyze the reverse effects of work on family, both have overlooked how work and family trajectories are interrelated across longer time windows of the life course. A recent line of research has conceptualized work-family trajectories as interlocked multidimensional life course processes and has examined them jointly.

In the US context, Han and Moen (1999) propose a couple-careers model based on the life course perspective to examine work and family trajectories jointly for men and women. The authors use sequence analysis to identify five distinctive career pathways and find that those featuring “delayed-entry” and intermittent career were followed exclusively by women while men were more likely to follow “orderly” and “fast-track” career pathways. Using a marital stability score constructed from a detailed marital history to capture the family trajectory, the authors then show that women in upwardly mobile career paths are more likely to experience marital instability, but men tend to reap the benefits of both career mobility and stable marriage. Using a qualitative case example to illustrate the negotiation of work and family within couples, one husband acknowledged that his wife “sacrificed her career in favor of [their] being able to live here” (p. 107).

Aisenbrey and Fasang (2017) use sequence analysis to examine how gender inequality in work-family trajectories unfold from early adulthood until middle age in two different welfare state contexts, the US and Germany. The authors demonstrate that patterns of work-family interplay across the life course in the United States are less gendered overall but differ widely by social class. On the upper end of the social ladder, work-family patterns characterized by high occupational prestige are fairly equally accessible for men and women. But this gender equality is only a white privilege that does not extend to black men and women. However, on the lower end, both white and black women are far more likely than men to experience the joint occurrence of single parenthood and unstable low-prestige work careers in the United States. The contrast by social class points to the disparate policy supports at the intersections of gender, class, and race in the United States, where disadvantaged lower-class minority women face cumulative pressure from both the labor market and family.

In the context of Germany, the authors find significant and strong gender effects for all work-family clusters. The combination of a high-prestige career with having a partner and several children is largely inaccessible to women. But the more extensive safety net of the German social security system prevents highly unstable low-prestige work trajectories, precluding the coupling between low-prestige employment and single parenthood in Germany. More recently, Fasang and Aisenbrey (2021) examine intersectional inequalities in work and family life courses by gender and race in the U. S. context. Using data from the NLSY79, the study reveals gendered and racialized constraints for Black women who experience the strongest interdependence between work and family life and instability spillovers between the two life domains.

Davia and Legazpe (2014) depict the joint trajectories of employment and fertility for women in Spain over a 20-year span from age 16–35. Using optimal matching analysis, they identify four patterns: (1) early marriage/non-working mother/high fertility; (2) late marriage/working mother/low fertility; (3) early marriage/working mother/high fertility; and (4) late marriage/low labor force participation/low fertility. The authors then demonstrate that education, as a human capital indicator, predicts trajectories featuring higher attachment to the labor market and low fertility. They find some evidence that those who have more traditional values are found in more traditionally oriented trajectories (i.e., early marriage, low labor force attachment, and high fertility).

Scherger et al. (2016) study the joint trajectories of work and family in cohorts of individuals born in the first half of the 20th century in the UK. They demonstrate that while the majority of men followed a trajectory of marriage and family formation with a relatively continuous career, the family-work trajectories of women varied noticeably from one cohort to the next, including increased labor market participation combined with fewer and shorter breaks from work to care for children.

Van Winkle and Fasang (2021) use data collected across Europe to quantify changing employment and family patterns from ages 18 to 50 and to decompose the variance in work and family lives attributable to cohort and country. The authors find a negligible increase in family complexity and a moderate increase in employment complexity over the life course across cohorts, which is in stark comparison to large and stable cross-national differences in work and family lives across 30 European countries. Their findings reveal polarization in Europe between most Eastern and Southern European countries with stable low family complexity compared to Nordic and some Western European countries with high and increasing family complexity. But their study considers work and family trajectories as parallel life trajectories without modeling the two domains jointly.

Studies conducted in non-Western societies to examine work and family trajectories are relatively scarce. Rindfuss et al. (2010) examine the temporal sequence of important life events in family and employment in Japan and find that the life course sequences of young Japanese men and women are very orderly, in contrast to the macro changes in the economy and population. Even though changes did occur in the family sphere, they feature an orderly postponement of both marriage and employment for both men and women; divorce and non-marital childbirth are rare while increasing. Clark and Yi (2020) find similar family patterns in China: while young adults in China postpone family formation as seen in increasing age at marriage, marriage is still the norm and having a child takes place quite rapidly after marriage. Overall, their analysis suggests more continuity than change in Chinese young adults' life course decisions with respect to family formation. Using comparable survey data across 63 low- and middle-income countries, Pesando et al. (2021) adopt sequence analysis to examine shifting family trajectories during transition to adulthood for women across countries and cohorts, documenting significant differences in life course patterns of sexual intercourse, union, and childbirth by macro-regions yet relative stability across cohorts.

## 5. Discussion

Life course principles, induced from Elder's (1974) classic study of the long-term influences of early childhood experience in the Great Depression on life course outcomes, highlight the value of studying personal biography within contexts defined by historical time and place. Recent study of stratification and mobility has been embracing the life course paradigm with its emphasis on trajectories, rather than the point-in-time measures of attainment in traditional mobility research. With the increasing availability of longitudinal survey data and advanced statistical techniques, transitions and trajectories have been modeled in multiple substantive areas to better understand the dynamic processes underlying individual outcomes. Trajectories of intragenerational career mobility have been examined along both continuous and categorical dimensions. Research also documents divergent career mobility trajectories across social groups, especially the prominent gender gap. The interplay of family and work informs important differences in the life course trajectories of men and women, as the motherhood penalty shapes women's disadvantaged career trajectories in the long run.

In conclusion, we would like to draw attention to gaps in the literature and potentially fruitful directions for future research on the intersections of work and family lives. The continuing emphasis of the life course theoretical tradition on individual “agency” has received relatively little attention in recent empirical studies of work and family trajectories that are cited in this review (Bernardi et al., 2019; Heckhausen et al., 2019). Early groundbreaking work from life course social psychology highlights the significant impacts of agentic resources on a diverse range of life course outcomes, but recent studies of life course transitions and trajectories of work and family cited in this review have largely focused on macro-level structural constraints and inequalities across social groups without incorporating the role of agency. The paucity of measures of agentic resources in nationally representative surveys, such as aspirations, plans, values, optimism, and self-efficacy, is a severe impediment to understanding the psychological micro-dynamics underlying intergenerational and intragenerational mobility. Understanding the processes underlying shifts in agency would lead to better understanding of the micro-level forces behind upward and downward mobility trajectories. While this review highlights quantitative studies, we acknowledge that qualitative studies of the work and family life course have contributed greatly to the understanding of subjective dimensions of work-family linkages and transitions. Future work should build on the impressive pioneering studies by Rubin (1976), Damaske (2013), and Moen (2016b) among others.

Further incorporation of a second major principle of life course analysis into mobility studies, that of “time and place,” would likewise enhance understanding of work and family transitions and trajectories, their interrelations over time, and their long-term impacts on individual wellbeing.

Research on mobility has neglected some historical trends with profound implications for work and family. Increasing family diversity is a case in point. Societies, particularly in the United States and Western Europe, have experienced increasing rates of cohabitation, divorce, remarriage, and the expansion of marriage rights to non-heterosexual couples (Lesthaeghe, 2010). Contemporary research on work and family careers is focused near exclusively on the families of heterosexual men and women (Baumle and Poston, 2011; Castro Varela and Bayramog, 2021). Also neglected in the literature is the historical increase in human longevity and its repercussions in work and family lives (Moen, 2016a). Research on increasingly diversified work and family trajectories after retirement and their impacts on the wellbeing of the aging population would benefit from collaboration between life course and mobility researchers.

As described in the introduction, life transitions and trajectories in work and family are becoming more complex due to broad historical change, including technological development, growing economic precarity, changing gender attitudes and roles, and increasing migration within and across national borders. As also noted, extant research has mainly been conducted in the United States and Western Europe. Future research should address the potential impacts of rapid historical shifts in different societies, especially in the Global South and Low- and Middle-Income Countries in general, and how they may account for divergent national trends in work and family life. Comparative studies should also assess institutional differences and changes across countries throughout the world that could account for their distinct responses to historical change, with some protective of stable work and family careers, and others exacerbating its disruptive potential. These include structural factors like the character of the school to work transition, the level of employment protection, safety net provisions, unionization, taxation policies, retirement benefits, etc., as well as cultural variation, as enshrined in family law and the relative emphasis on individualism and collectivism.

The implications of recent cataclysmic change on life course trajectories also deserve attention, including the interrelated problems of climate change, global pandemic, war, large-scale migration, and economic upheaval. These are likely to have broad implications for future inter- and intra-generational mobility trajectories. Attention to long-term historical shifts as well as these potentially catastrophic challenges, along with continuing progress in the development of analytic methods and longitudinal data sources, will yield enhanced understanding of work and family trajectories throughout the life course.

## Author contributions

XH wrote the entire initial draft of the review, constructed the references list, and formatted the final manuscript. JM's comments guided the development of parts of the initial draft. Both authors contributed to revisions in response to reviewers' comments and approved the final version.
